# Closing the rural diabetes research gap: a case for coordinated international collaboration

**DOI:** 10.1080/16549716.2026.2731493

**Published:** 2026-09-21

**Authors:** Giuliana Murfet, Wenyong Wang, Rahul D. Barmanray, Ashley H. Ng, Mette Juel Rothmann, Natalie Wischer, Freda Mold, Grant D. Brinkworth, Wenbo Peng, Karen Pearce, Nicola Carey

**Affiliations:** a School of Nursing, UTAS Health, University of Tasmania, Burnie Tasmania, Australia; b Queensland Digital Health Centre, The University of Queensland, Herston, QLD, Australia; c Department of Medicine, The Royal Melbourne Hospital, The University of Melbourne, Melbourne, Australia; d Monash Centre for Health Research and Implementation, Monash University, Clayton, Australia; e Steno Diabetes Center Odense, Odense University Hospital, Odense, Denmark; f Department of Clinical Research, Faculty of Health Science, University of Southern Denmark, Odense, Denmark; g National Association of Diabetes Centres, Australian Diabetes Society, Sydney, Australia; h School of Health Sciences, Faculty of Health and Medical Sciences, University of Surrey, Guildford, United Kingdom; i Health Economics & Research Strategic Programs and Innovation, Diabetes Australia, Milton, Queensland, Australia; j School of Public Health, Faculty of Health, University of Technology Sydney, Sydney, Australia; k School of Nursing, Auckland University of Technology, Auckland, New Zealand

**Keywords:** Rural health, health equity, diabetes mellitus, nursing, research consortium

## Abstract

Rural, regional and remote communities experience a disproportionate burden of diabetes and persistent barriers to specialist care, including workforce shortages, geographic isolation, transport costs, fragmented services and uneven digital infrastructure. However, much of the evidence guiding diabetes service design is derived from urban settings, while rural studies are often localised, short-term and difficult to compare or scale. This article argues that addressing rural diabetes inequity requires coordinated international research rather than isolated projects or the transfer of urban models into rural contexts. It introduces the International Consortium for Rural Diabetes Research (ICRDR), an international collaboration of clinicians, researchers, health service leaders and diabetes stakeholders established to align research priorities, develop shared methodologies and outcome measures, and support multicentre and cross-jurisdictional studies. The ICRDR will initially develop community-informed research priorities involving people with lived experience, clinicians, researchers, policymakers and funders. Its longer-term agenda includes evaluating contextually appropriate models of care, digital health approaches, workforce capability and connections between primary and specialist care and supporting research capacity in low- and middle-income countries. Coordinated international research infrastructure can strengthen the relevance, implementation and scalability of rural diabetes research and support the translation of evidence into more equitable and sustainable models of care.

## Paper Context

● **Main findings**: Rural diabetes research remains fragmented and often relies on evidence generated in metropolitan health systems, highlighting the need for coordinated international rural research infrastructure.

● **Added knowledge**: This paper describes how the International Consortium for Rural Diabetes Research can support shared priorities, stronger methods, multicentre studies and community-informed research.

● **Global health impact for policy and action**: Governments, funders and health services should invest in sustained rural diabetes research infrastructure and involve rural communities, clinicians and people living with diabetes in setting priorities and redesigning care.

## Background

Rural diabetes inequity is evident across both high-income and low- and middle-income countries [1]. Definitions of rurality and remoteness vary across countries and health systems and may incorporate population density, settlement size, geographic distance and access to services. In this article, rural and remote settings broadly encompass communities classified as non-metropolitan or geographically less accessible according to relevant national or jurisdictional frameworks [2]. Despite their heterogeneity, these communities commonly experience geographic isolation, long travel distances, workforce shortages, intermittent specialist access, limited local service capacity and uneven digital infrastructure, which can affect the accessibility, continuity and quality of diabetes care [3,4]. Across countries, people living in rural, regional and remote areas consistently experience a disproportionate burden of diabetes and reduced access to specialist care compared with those in urban areas [3,4]. The International Consortium for Rural Diabetes Research (ICRDR) has been established in response to this gap. It is an international collaboration of clinicians, researchers, health service leaders and diabetes stakeholders across Australia, New Zealand, the United Kingdom and Nordic regions, with plans to expand participation to low- and middle-income countries, including regions such as India, sub-Saharan Africa and the Pacific Islands, to strengthen cross-country learning and address rural diabetes inequities across diverse health systems. This article argues that closing the rural diabetes research gap requires coordinated international collaboration rather than isolated local projects or the transfer of urban models into rural settings.

The impact of these access barriers is evident internationally [1]. Diabetes prevalence is approximately 30% higher and diabetes-related hospitalisation rates more than twice as high in remote Australia [5]. In the United States, diabetes prevalence is also higher among rural than urban adults [6], while across 42 low- and middle-income countries, rural populations with diabetes are 15–30% less likely to receive recommended diagnosis and treatment, and 14% less likely to achieve target glycaemia [1]. These inequities may be particularly pronounced among Indigenous Peoples and other underserved rural populations, although experiences and health-system contexts vary across countries [7]. They are shaped by workforce shortages, intermittent specialist access, transport costs, lower health service density, digital exclusion, socioeconomic disadvantage, health literacy and trust in health services [8,9]. Rural diabetes care therefore needs models that improve access, continuity and quality while fitting local care settings [8,10].

Rural diabetes care operates under different conditions, not simply with fewer resources [4,8]. People with diabetes and their families may travel long distances for care, creating costs, time away from work and loss of income [11,12]. Specialist services may be intermittent, visiting or concentrated in regional centres, making access logistically difficult [3,4]. Continuity can be fragile, particularly for people with complex needs, pregnancy, advanced technology needs or complications requiring multidisciplinary input [13,14]. These challenges differ across diabetes types: Type 1 diabetes often requires reliable specialist input and technology support, while Type 2 diabetes models must address high population burden, comorbidity and primary care capacity [15,16]. Primary care may carry work otherwise shared with specialist services without equivalent expertise, infrastructure or capacity, even within the same country [8].

## Rural diabetes research gap

Much of the evidence guiding diabetes service design has been developed in urban health systems [8,9]. Large trials, implementation studies, and service redesign initiatives remain predominantly shaped by urban health systems [17,18]. Although larger studies and audits may include rural participants, fewer are designed to generate rural-relevant evidence or evaluate rural diabetes systems [19]. This limits external validity: metropolitan interventions may assume stable workforces, nearby specialists, reliable transport, strong digital infrastructure or capacity for frequent reviews [9]. Rural inequity therefore requires fit-for-purpose models designed for and tested within rural contexts [9]. Rural populations should not remain peripheral to the research that informs their care [9].

Rural diabetes studies often remain local, short-term or focused on one component of care rather than system-level reform [19]. The Medical Research Council guidance (2021) for complex interventions emphasises context, programme theory, stakeholder engagement, key uncertainties, intervention refinement, implementation and economics [20]. For rural diabetes care, this means asking how models work, for whom and under what conditions, and whether they can be adapted and scaled across diverse settings [20,21]. Cultural and contextual differences, and implementation barriers and facilitators, are central to adoption and sustainability [22].

Digital health has considerable potential to improve rural diabetes care by supporting access, continuity and care coordination [9]. Telehealth can reduce travel, remote monitoring can support earlier clinical review and monitoring, electronic medical records can improve information sharing, and decision support tools may assist timely care [9,23]. These benefits are particularly relevant for diabetes, which requires regular monitoring, medication adjustment, complication screening, education and multidisciplinary co-operation [9,24]. However, effective use requires reliable connectivity and access to suitable devices, interoperable systems, workable clinical workflows, clinician adoption, appropriate governance, patient access, digital literacy and sustainable support [9,25,26]. Digital health is therefore an enabler rather than a complete solution and must be adapted to local infrastructure, workforce and patient needs [9,25,27].

Similar challenges affect rural diabetes models of care [10,28–30]. Outreach clinics, telehealth-supported review, shared care, advanced practice roles, multidisciplinary partnerships and hub-and-spoke models are often developed in response to local needs rather than through deliberate design and comparative evaluation [10,28–30]. They may be implemented without shared frameworks, coordinated evaluation or system-level oversight [10,28–30]. Rural care can therefore remain dependent on local initiatives that are difficult to compare, sustain or scale [19]. Workforce shortages, delayed access to specialist care, fragmented services, transport barriers, uneven digital infrastructure and challenges sustaining multidisciplinary care are common across Australia, New Zealand, the United Kingdom, Nordic regions, Canada and elsewhere [9,19].

## The role of the ICRDR

Related initiatives are gaining momentum. Recent rural diabetes research has examined barriers to research participation among adults with Type 2 diabetes, including concerns that researchers may ‘fly in’ to collect information without creating lasting local benefit [10,23,31,32]. Conversely, community-engaged work in rural regions with people with Type 1 diabetes has demonstrated the value of rural stakeholder research teams and person-centred research agendas [32]. However, existing work remains largely project-based, condition-specific, or nationally situated [19]. To our knowledge, there is no established international rural diabetes research consortium focused on coordinating priorities, shared methods, comparative implementation research, and translation into rural models of care. Table 1 summarises the key challenges described above and how coordinated international collaboration could respond.

**Table 1.** Limitations in the current rural diabetes research landscape and potential responses through coordinated international collaboration.

Limitations	Why this matters	ICRDR response
**Urban-centred evidence base** Much of the evidence guiding diabetes service design has been developed in urban health systems. Fewer studies are designed to generate rural-relevant evidence or evaluate rural diabetes systems.	Metropolitan interventions may assume stable workforces, nearby specialists, reliable transport and stronger digital infrastructure, limiting external validity for rural settings.	Support research designed for rural contexts and enable comparative studies across diverse rural settings.
**Localised and short-term research** Rural diabetes studies often remain local, short-term or focused on one component of care rather than system-level reform.	This limits transferability, long-term learning and understanding of how models work across settings.	Enable multicentre and cross-jurisdictional studies and more coordinated evaluation.
**Variable definitions of rurality and remoteness** Definitions vary across countries and health systems and may incorporate population density, settlement size, geographic distance and access to services.	Variation reduces comparability across studies and jurisdictions.	Establish shared language and more consistent ways of describing rural settings.
**Limited coordinated evaluation and comparable measures** Models of care are often developed in response to local needs without shared frameworks, coordinated evaluation or system-level oversight.	Local initiatives become difficult to compare, sustain or scale.	Develop shared methodologies, comparable measures and coordinated data collection.
**Limited implementation and context-specific evidence** Rural diabetes care needs models that improve access, continuity and quality while fitting local care settings. Research should ask how models work, for whom and under what conditions, and whether they can be adapted and scaled.	Without this understanding, promising models may not be workable or sustainable in real-world rural contexts.	Support comparative implementation research that is grounded in context, and examines transferability and scalability.
**Persistent structural barriers** Rural settings commonly experience geographic isolation, long travel distances, workforce shortages, intermittent specialist access, limited local service capacity and uneven digital infrastructure.	These barriers affect the accessibility, continuity and quality of diabetes care.	Prioritise research on contextually appropriate models of care, digital health, workforce capability, and links between primary and specialist care.
**Fragmented international landscape** Existing work remains largely project-based, condition-specific or nationally situated. To our knowledge, there is no established international rural diabetes research consortium focused on coordinating priorities, shared methods, comparative implementation research and translation into rural models of care.	Fragmentation limits shared learning, comparable evidence and translation into practice.	Provide a structure for community-informed priorities, shared methods, collaborative studies and cross-jurisdictional partnerships.

The ICRDR provides a structure for coordinated research across countries facing similar challenges. It aims to align priorities, build trusted partnerships and support multicentre and cross-jurisdictional studies. Its value lies in enabling shared methods, comparable outcomes, coordinated data collection and comparative analysis across diverse rural settings. These barriers are too complex for isolated local studies alone. Coordinated infrastructure can strengthen governance, improve translation into real-world care and increase competitiveness for major funding. It can also unite expertise in diabetes care, digital health, health services research, workforce redesign, implementation science, and health economics and equity.

The ICRDR’s first contribution will be agenda-setting through the development of community-informed research priorities. Drawing on established approaches such as James Lind Alliance-style priority setting and Delphi consensus methods [33,34], the Consortium will bring together people with lived experience, clinicians, health system leaders, researchers, policy stakeholders, and funders to identify the most urgent pain points, unmet needs, and practical barriers across rural communities. Research priorities should not be determined only by academic interest or technological possibility. They should be grounded in the problems that people with diabetes, clinicians, and health systems experience as most urgent and consequential.

This work will position the ICRDR to set a credible, community-informed and internationally relevant agenda. Studies should test which models improve outcomes, which digital approaches are workable and sustainable, how rural workforce capability can be strengthened, how specialist and primary care can be connected and how findings can inform service redesign. The Consortium should also establish shared language, comparable measures, governance pathways and academic-health service partnerships. Early success can be assessed through network reach and diversity, clinician and consumer engagement, agreed priorities, joint protocols, shared data elements and multisite collaborations. Formal governance, working groups, shared operating processes and a pipeline for collaborative grants and multicentre studies will support larger trials and coordinated evaluation. Over its first 5 years, the ICRDR anticipates progressing from establishing and expanding the international network and developing shared research priorities and approaches, to strengthening consumer, community and research engagement and progressing collaborative multicentre research and implementation studies. This broad trajectory is intended to remain flexible, allowing the Consortium to adapt as priorities, partnerships and funding opportunities develop.

## Conclusion

The case for coordinated rural diabetes research is compelling. Rural diabetes inequity is well established, the burden is substantial, and current research approaches are insufficient. Continued reliance on urban evidence, local pilots and fragmented innovation are unlikely to deliver the required change. Sustained investment in coordinated rural diabetes research infrastructure is needed to set shared priorities, support multicentre studies, strengthen research capacity in low- and middle-income countries, and translate evidence into service redesign. The ICRDR is one practical response. Rural communities, clinicians and people living with diabetes must have a central role in setting priorities and redesigning future care.
